# Prediction of long-term segmental and global functional recovery of hibernating myocardium after revascularisation based on low dose dobutamine and late gadolinium enhancement cardiovascular magnetic resonance

**DOI:** 10.1186/s12968-014-0083-z

**Published:** 2014-10-03

**Authors:** Sigita Glaveckaite, Nomeda Valeviciene, Darius Palionis, Roma Puronaite, Pranas Serpytis, Aleksandras Laucevicius

**Affiliations:** Department of Cardiovascular Medicine, Vilnius University; Centre of Cardiology and Angiology, Vilnius University Hospitals Santariskiu Klinikos, Santariskiu str. 2, 08661 Vilnius, Lithuania; Department of Radiology, Nuclear Medicine and Physics of Medicine, Vilnius University; Centre of Radiology and Nuclear Medicine, Vilnius University Hospitals Santariskiu Klinikos, Vilnius, Lithuania

**Keywords:** Cardiovascular magnetic resonance, Late gadolinium enhancement, Contractile reserve, Revascularisation, Functional improvement, Follow-up studies

## Abstract

**Background:**

This study sought to evaluate the relation between long-term segmental and global functional outcome after revascularisation in patients with chronic ischaemic left ventricular dysfunction (LVD) and baseline markers of viability: late gadolinium enhancement (LGE) transmurality and contractile reserve (CR).

**Methods:**

Forty-two patients with chronic ischaemic LVD underwent low-dose dobutamine- (LDD) and late gadolinium enhancement (LGE) cardiovascular magnetic resonance (CMR) before surgical or percutaneous revascularisation. Regional and global left ventricular (LV) functions and LGE were repeatedly assessed 6 ± 1 and 35 ± 6 months after revascularisation. In total, 319 at baseline dysfunctional and successfully revascularised segments were available for statistical analysis.

**Results:**

The likelihood of long-term functional improvement was directly related to the presence of CR and inversely related to both the LGE and the degree of contractile dysfunction at baseline. The time course of functional improvement was protracted, with significantly more delay in segments with more extensive LGE (p = 0.005) and more severe contractile dysfunction at baseline (p = 0.002). The presence of CR was the predictor of earlier functional improvement (p < 0.0001). Using a definition of viable segment as a segment without any LGE or with any LGE and producing CR during LDD stimulation, ≥55% of viable segments from all dysfunctional and revascularised segments in a patient was the only independent predictor of significant improvement (≥5%) in the left ventricular ejection fraction (LVEF) after revascularisation, with a 72% sensitivity and an 80% specificity (AUC 0.76, p = 0.014). Reverse LV remodelling was observed in patients who had a significant amount of viable myocardium successfully revascularised.

**Conclusions:**

In patients with chronic ischaemic LVD, improvement of dysfunctional but viable myocardium can be considerably delayed. Both the likelihood and the time course of functional improvement are related to the LGE, CR and the degree of contractile dysfunction at baseline. At 35 ± 6 months after revascularisation, patients with ≥55% of viable segments from all dysfunctional and revascularised segments significantly improve LVEF and experience reverse LV remodelling. A combination of LDD–CMR and LGE–CMR is a simple and powerful tool for identifying which patients with impaired LV function will benefit from revascularisation.

## Background

Hibernating myocardium is normally defined as viable and dysfunctional myocardium that improves in function with restoration of adequate blood flow following revascularisation [1]. This reversible state should be clearly distinguished from irreversibly injured or infarcted myocardium, in which case the restoration of coronary blood flow would not be justified. The current role of viability testing remains the prediction of potential functional and clinical improvement in patients with impaired LVEF, thereby facilitating a better estimate of the potential benefit of revascularisation therapy versus its risks [2]. However, three prospective randomized trials—the Heart Failure Revascularisation (HEART) Trial, the Positron emission tomography (PET) And Recovery following Revascularisation (PARR-2) trial, and the Surgical Treatment for Ischaemic Heart Failure (STICH) trial—have challenged this concept, as none found benefit for the use of viability testing in guiding management decisions or influencing mortality outcome [3-6]. The HEART trial terminated prematurely and was not sufficiently powered to draw conclusions [3]. The PARR-2 trial did not demonstrate a benefit from PET-guided management compared with standard care, although analysis of only those patients that did adhere to the PET-guided recommendations did reveal a significant mortality benefit [4]. The STICH trial viability sub-study found that viability testing did not alter outcomes (irrespective of management strategy) [6]. However the latter sub-study had several important methodological limitations [6]. Even after the STICH trial, there is still a need for prospective studies designed to clarify whether revascularisation of a significant amount of hibernating myocardium is beneficial compared with optimal medical therapy.

Cardiovascular magnetic resonance (CMR) with its high spatial resolution, provides qualitative and quantitative, global and regional information on myocardial anatomy and function. In combination with a gadolinium-based contrast agent, CMR allows an accurate quantification of the myocardial scar [7] and predicts the likelihood of functional recovery after revascularisation [8-11]. The cut-off value of LGE used directly influences the technique’s accuracy for predicting functional recovery. As the cut-off value for LGE increases, the sensitivity falls but specificity rises. For example, >75% LGE has a 100% negative predictive value (NPV) for functional recovery after revascularisation [8]. However, in patients with <75% LGE, the additional assessment of CR by LDD-CMR improves the predictive accuracy over LGE imaging alone [11-13]. Based on these insights, there is a rationale to combine CMR-based viability parameters in order to better predict improvement in dysfunctional myocardial segments after revascularisation [11,14,15].

In most studies functional outcome is assessed 3–6 months after revascularisation, whereas a longer follow-up interval would be more appropriate, taking into account that functional recovery may be considerably delayed in hibernating myocardium having more advanced structural damage [10]. So far, few studies have investigated the long-term functional outcome and time course of functional recovery in relation to baseline markers of viability [10,16-19]. To the best of our knowledge, the above-mentioned studies didn’t explore CR and LGE, both assessed by CMR as predictors of long-term segmental and global left ventricular functional recovery.

To address the above-mentioned issues, we used CMR to study a group of patients with chronic ischemic LVD before and 6 ± 1 and 35 ± 6 months after revascularisation. The primary goal of our study was to assess the long-term functional outcome of left ventricular segmental and global function after revascularisation in relation to baseline markers of viability – CR and LGE. The second goal of our study was to determine the optimal predictor of significant (≥5%) improvement in LVEF at the end of study period.

## Methods

The methods have been described in detail before [12].

### Patients and study design

A prospective evaluation of CMR based markers of viability was performed in 42 patients with LVD (LVEF 36 ± 8%) before they underwent either surgical (n = 32) or percutaneous (n = 10) revascularisation. Three CMR scans were performed for all study patients: a baseline (first) CMR scan 12 (range 2 – 33) days before revascularisation, a second CMR scan 27 ± 4 weeks (6 ± 1 months) after revascularisation and a third CMR scan 151 ± 27 weeks (35 ± 6 months; median 2.9 years, range 1.5 – 4.0 years) after revascularisation. The aim of CABG or PCI was to obtain complete revascularisation, which was technically possible and performed in all study patients. The short-term (6 months after revascularisation) data on forty six consecutive patients meeting the following inclusion criteria: (1) coronary artery disease (>70% stenosis in one or more major epicardial vessels), scheduled for a revascularisation procedure; (2) LVEF ≤45%; (3) at least two adjacent segments with wall motion abnormalities at rest; (4) no infarction or revascularisation within the last two months; and (5) no contraindications to CMR (e.g., a pacemaker), has been published previously [12]. Of the 4 patients who did not complete the whole study, 2 had pacemakers or defibrillators implanted during the period between follow-up CMR scans, 1 decided not to undergo the third CMR scan because of a severe disabling condition related to cerebral infarction, and 1 experienced sudden cardiac death in the period between follow-up CMR scans. None of the patients were excluded from the study for technical reasons or image quality. The baseline characteristics of the 42 patients who underwent all three CMR scans are listed in Table 1. All patients were in stable clinical condition at the time of the CMR scans and there was no clinical evidence of ischemic events during the period between the revascularisation and the third CMR scan. After revascularisation, all patients received standard pharmacological treatment for heart failure, as per current recommendations [20].

**Table 1:** **Baseline characteristics of the study patients**

**Patient characteristics**	
Males/females, *n*	39/3
Age, years	65 ± 10
Risk factors, %	
Systemic hypertension	98
Diabetes mellitus	26
Hypercholesterolemia	83
Smoking	29
Positive family history	29
Coronary angiography, %	
Single-vessel disease	7
Two-vessel disease	14
Three-vessel disease	79
History of MI, %	88
Previous revascularisation, %	
CABG	2
PCI	33
Ejection fraction at baseline assessed by CMR, %	36 ± 8
Days between baseline CMR and revascularisation (range)	12 (2 – 33)
Treatment, %	
Beta blockers	86
Angiotensin-converting enzyme inhibitors	71
Nitrates	69
NYHA functional class ≥ III, %	79

The study was approved by the Lithuanian Bioethics Committee (No. 158200-13-576-178) and informed written consent was obtained from each patient prior to inclusion in the study.

### CMR protocol

All the CMR scans were performed using a 1.5 Tesla MR scanner (Avanto, Siemens Medical Solutions, Erlangen, Germany) with the patient in a supine position, using prospective electrocardiographic (ECG) gating. Steady-state free precession cine CMR was performed while the breath was held, and 4-, 3- and 2-chamber views, as well as a short axis stack covering the left ventricle every 8 mm without a gap, were acquired at rest and then after each dose of dobutamine (5 and 10 μg/kg/min) (TE/TR/flip angle 1.22 ms/63 ms/65 degrees, field of view (FOV) 250 mm, voxel size 1.9 × 1.3 × 8 mm^3^, matrix size 109 × 192). After revascularisation, only rest images were acquired using the same technique.

Ten to fifteen minutes after infusing 0.15 mmol/kg of a commercially available gadolinium-based contrast agent (gadopentetate dimeglumine or gadodiamide), an inversion recovery gradient-echo sequence triggered to end-diastole (TE/TR/flip angle 3.2 ms/700 ms/25 degrees, FOV 400 mm, matrix size 156 × 256 mm, and a typical voxel size of 2.1 × 1.6 × 8 mm^3^) was performed with an inversion time (240 to 330 ms) chosen to reduce the signal from normal myocardium. Angulation was kept constant for the short-axis and LGE imaging to enable a match between the LGE and wall motion images. LGE imaging was performed before revascularisation and was repeated 6 ± 1 and 35 ± 6 months after revascularisation in order to exclude from the study patients having significant periprocedural injury (new LGE zones on second CMR scan) or significant myocardial injury at follow-up (new LGE zones on third CMR scan).

### Post-processing analysis

We analysed the cine images and contrast-enhanced images using a model in which the LV was divided into 17 segments [21]. The wall motion was graded as 1 (normal), 2 (mild hypokinesia), 3 (severe hypokinesia), 4 (akinesia) or 5 (dyskinesia) by 2 blinded investigators. For the patients undergoing percutaneous revascularisation, segments were considered to be undergoing revascularisation according to the scheme suggested by *Haug* [22]. The LV apical segment was assigned to a specific coronary artery territory according to the vessel anatomy on a conventional angiogram. For the global LV functional analysis, all short-axis slices from the base to the apex were analysed with Argus software (Siemens) by two independent experienced observers (with certification of Level 2 competency in CMR and more than 5 years of work experience in a CMR unit). Manual tracing of the left ventricular endocardial borders of successive short-axis slices at end-diastole and end-systole was performed (papillary muscles were excluded from the volume calculations) in order to calculate end-diastolic volume (EDV), end-systolic volume (ESV), stroke volume (SV) and left ventricular ejection fraction (LVEF). All LV volumes were indexed to body surface area. The sphericity index (SI) was measured by dividing the length of the LV from the apex to the mitral annulus by the width of the LV at the basal aspect of the papillary muscles in the end-diastolic apical four-chamber view. The wall motion score index (WMSI) was calculated by dividing the sum of the scores by the number of segments per patient. The mitral valve regurgitant fraction (MV RF) is the ratio of the mitral regurgitant volume divided by the LV SV (where the mitral valve regurgitant volume is the difference between the LV SV and the aortic forward stroke volume). An absolute change in LVEF ≥5% 35 ± 6 months after revascularisation was considered to be significant based on previously reported data about the effect of CABG on LV function [9], and taking into account intra- and interobserver variability in LVEF measurements by CMR [23,24]. When predicting significant LVEF improvement, a segment was considered viable if it either had no LGE or had any LGE and produced CR during LDD stimulation. The number of viable segments divided by the total number of dysfunctional and revascularised segments in a patient was expressed as a percentage that was used to predict significant LVEF improvement. We compared two groups: responders (i.e., patients with significant LVEF improvement) and nonresponders (i.e., patients without significant LVEF improvement (improvement of LVEF < 5%)). The extent of LGE within each segment was also measured by the two independent experienced investigators on short-axis, contrast enhanced CMR images. Contrast enhanced pixels were defined as those with image intensities > 2 SD above the mean image intensity in a remote myocardial region in the same image. LGE was assessed on a 5-grade scale [8] and analysed quantitatively by dividing the hyperenhanced area, as measured by Argus software-assisted tracings, by the total area in each segment before being expressed as a percentage. Dysfunctional and successfully revascularised segments without an increase in the LGE area on the second and third CMR scans were analysed. An improvement in wall motion at late follow-up of at least 1 grade, with the exception of improvement from grade 5 to grade 4 compared with baseline, was regarded as functional improvement or viability of the segment. The LDD-CMR was regarded as indicative of viability or CR when there was an improvement of 1 wall motion grade at either the 5 or 10 μg/kg/min dose. Interobserver and intraobserver agreement was assessed in 10 patients for the transmural LGE grading, improvement in segmental and global contractility and CR (Cohen’s *κ*, 0.82 – 0.88 interobserver and 0.86 – 0.89 intraobserver). All reviewers of segmental wall motion, CR, LGE and functional improvement were blinded to each other and to the clinical data of the patients. All discordant assessments were jointly reviewed.

### Statistical analysis

All values are expressed as mean ± standard deviation (SD). We used χ-squared tests to evaluate the trend in likelihood of segmental improvement in the different groups according to LGE, CR and baseline contractility. We used the 0% LGE and mild hypokinesia groups as references, so all other groups were related to those two. The logistic regression equation was used to calculate odds ratios, which expressed the likelihood of improvement relative to the functional outcome of segments without any LGE or CR or with mild hypokinesia.

In order to better evaluate the time course of segmental functional improvement we reclassified segments according to the time of improvement, dividing them into three groups: 1) segments with early (at 6 ± 1 months) improvement (this group incorporated segments with sustained improvement during the entire study period as segments with decreased segmental function at 35 ± 6 months), 2) segments with only late (at 35 ± 6 months) improvement, 3) segments without improvement. This reclassification was needed in order not to miss segments with early improvement despite a decrease in function at late follow-up. The χ-squared tests with Bonferroni method were used to evaluate the time course of segmental functional improvement in relation to the extent of myocardial scar and degree of contractile dysfunction at baseline.

The different baseline and follow-up characteristics of patients with and without significant improvement in LVEF 6 ± 1 months and 35 ± 6 months after revascularisation were compared. The values from both patient groups were expressed as mean ± SD. The effect of revascularisation was compared using a Wilcoxon signed-rank test. The continuous variables that were not distributed normally were compared by using a nonparametric test. The variables that differed significantly between groups were included in a forward stepwise (Wald) logistic regression analysis to determine the best independent predictor of significant LVEF improvement. A receiver operating curve (ROC) analysis was performed to validate the variables with the best predictive ability. The predictor of global functional recovery was treated as superior to the other methods if its area under the ROC curve (AUC) was significantly greater.

To ensure the statistical power of the prediction of segmental recovery, the required sample size of dysfunctional and successfully revascularised segments was calculated. For this purpose two logistic regression models were created in which recovery of segmental function was considered as the dependent variable and the independent variables were 50% threshold of either LGE (*β*_0_ = −0.754; *β*_1_ = 4.229) or CR (*β*_0_ = 0.995; *β*_1_ = 3.582). We calculated sample sizes for LGE and CR and considered the largest calculated sample size (n = 276) that guaranteed acceptable II type error in both models. All calculations were performed using IBM SPSS statistics software (version 21) and StAR [25] software. A p - value < 0.05 was considered statistically significant.

## Results

### Segmental LV function

At baseline, 714 segments (42 patients × 17 segments) were available for analysis. Almost 45% of these segments (n = 319) were considered dysfunctional and were successfully revascularised.

### Functional improvement in segmental function

**Figure 1:**
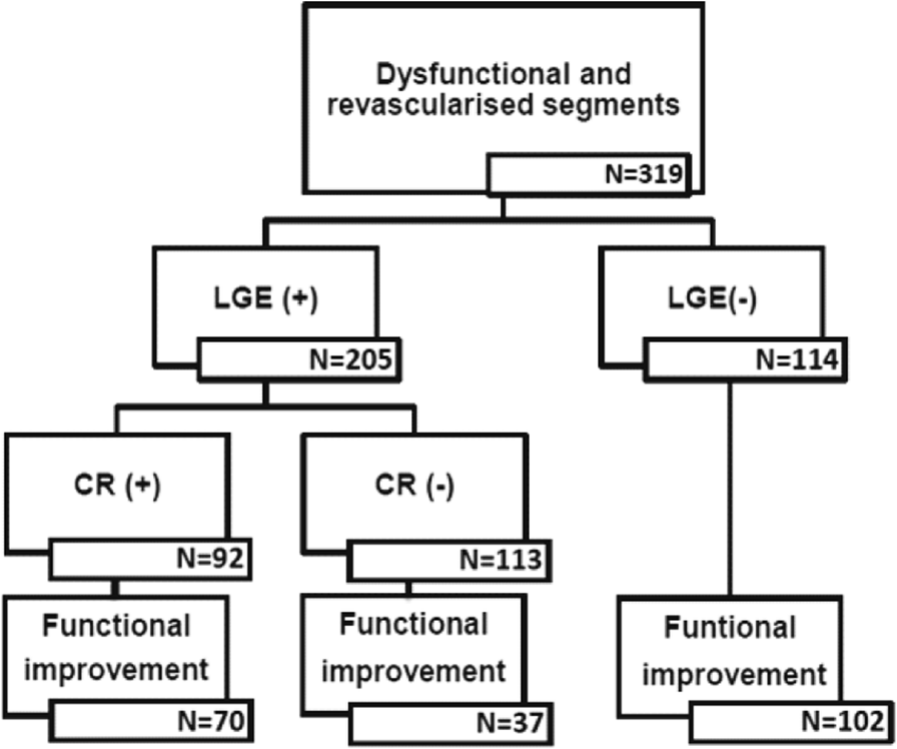
**Flow chart of analysed segments according to the presence of LGE, CR and late functional improvement at the end of the study period.**

At the end of the study period, functional improvement was observed in 209 (65.5%) segments, but the remaining 110 segments (34.5%) showed no signs of functional improvement (Figure 1). Compared with baseline, functional improvement was seen in 90, 86, 76, 47, and 15% of segments with no, 1–25%, 26–50%, 51–75%, and 76–100% LGE, respectively (Table 2). The likelihood of functional improvement was inversely related to the LGE during the entire follow-up: at the end of the study period, segments with 26–50%, 51–75%, and 76–100% LGE were 2.7 (1.3–5.8, p = 0.012), 9 (4.4–19.7, p < 0.0001), and 49 (18.1–130.5, p < 0.0001) times less likely to have functional improvement than segments without any LGE.

**Table 2:** **Functional improvement in segmental LV function at the end of the study period compared with baseline (segmental functional improvement is expressed in absolute numbers of improved segments and a percentage of baseline for every CMR scan according to LGE transmurality;** ***n*** **, number of dysfunctional segments at baseline)**

**CMR**	**LGE**				
	**0%**	**1-25%**	**26-50%**	**51-75%**	**76-100%**
**Baseline**	*n* = 114	*n* = 7	*n* = 82	*n* = 68	*n* = 48
**6 ± 1 months**	93 (81.6%)	5 (71.4%)	58 (70.7%)	29 (42.7%)	8 (16.7%)
**35 ± 6 months**	9 (7.9%)	1 (14.3%)	4 (4.9%)	3 (4.4%)	−1 (−2.1%)
**Total**	102 (89.5%)	6 (85.7%)	62 (75.6%)	32 (47.1%)	7 (14.6%)

The majority of the segments (157 of 185, 85%) with CR at baseline had functional improvement at the end of follow-up: segments with CR were 8.8 times (5.2 − 15.0, p < 0.0001) more likely to have functional improvement than segments without CR.

At the end of the study period, functional improvement was documented in 78% of the segments with mild hypokinesia, 54% in those with at least severe hypokinesia, and only 33% of those with akinesia or dyskinesia at baseline. Functional improvement was inversely related to baseline degree of segmental dysfunction during the entire follow-up: at 35 ± 6 months, segments with akinesia or dyskinesia at baseline were 4 times (2.1–7.7, p < 0.0001) less likely to have functional improvement than segments with mild hypokinesia.

Additionally, we compared the predictive values of CR, LGE50 (i.e. an LGE threshold value of 50%) and LGE50 + CR for long-term functional recovery in the same way as has previously been described in detail [12]. When the areas under the ROC curves are compared, the combined viability prediction model (LGE50 + CR) was significantly superior to CR alone in all the analysed sets of segments (AUC 0.78/0.72, respectively, p = 0.028), except the segments with an LGE from 26% to 75% (p = 0.345) and an LGE from 1% to 75% (p = 0.301). The combined viability prediction model (LGE50 + CR) was statistically significantly superior to the LGE50 alone in all the analysed sets of segments (p = 0.00001 in segments with any degree of LGE, p = 0.00007 in segments with LGE from 26% to 75% and p = 0.00013 in segments with LGE from 1% to 75%). The above-mentioned finding is consistent with our previously published short-term results [12] and shows the superiority of LDD–CMR over LGE–CMR in the prediction of short- and long-term segmental recovery, especially in segments with an LGE from 1% to 75%. Relying on this finding we used both the CR and the LGE for predicting whether a segment is viable or not.

### Time course of functional improvement

A total of 47 segments were dysfunctional at the end of the study period. Twenty-two (47%) segments improved at 6 ± 1 months but at the end of the study showed a decrease in segmental function, with six (27%) of these segments ending up at late follow-up with dysfunction of a lesser degree than at baseline. None of the revascularised normokinetic segments became dysfunctional at late follow-up. The long-term follow-up revealed worsening of segmental function in 5 (4%) initially normokinetic and nonrevascularised segments.

**Figure 2:**
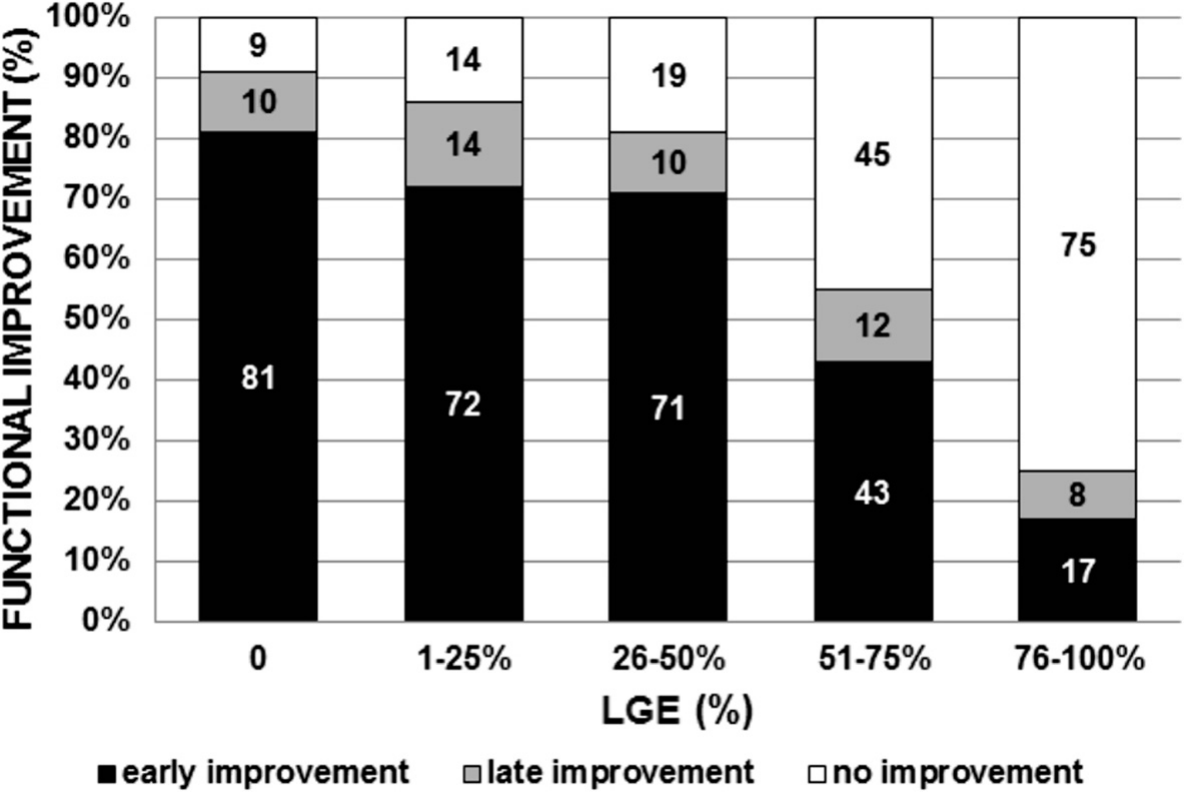
**Likelihood of functional improvement after revascularisation in relation to the time course and segmental LGE at baseline.** Early (at 6 ± 1 months; black bars) and late (at 35 ± 6 months; grey bars) functional improvement is expressed as a percentage of the total number of dysfunctional segments in each LGE category. All dysfunctional segments are included (n = 319).

**Figure 3:**
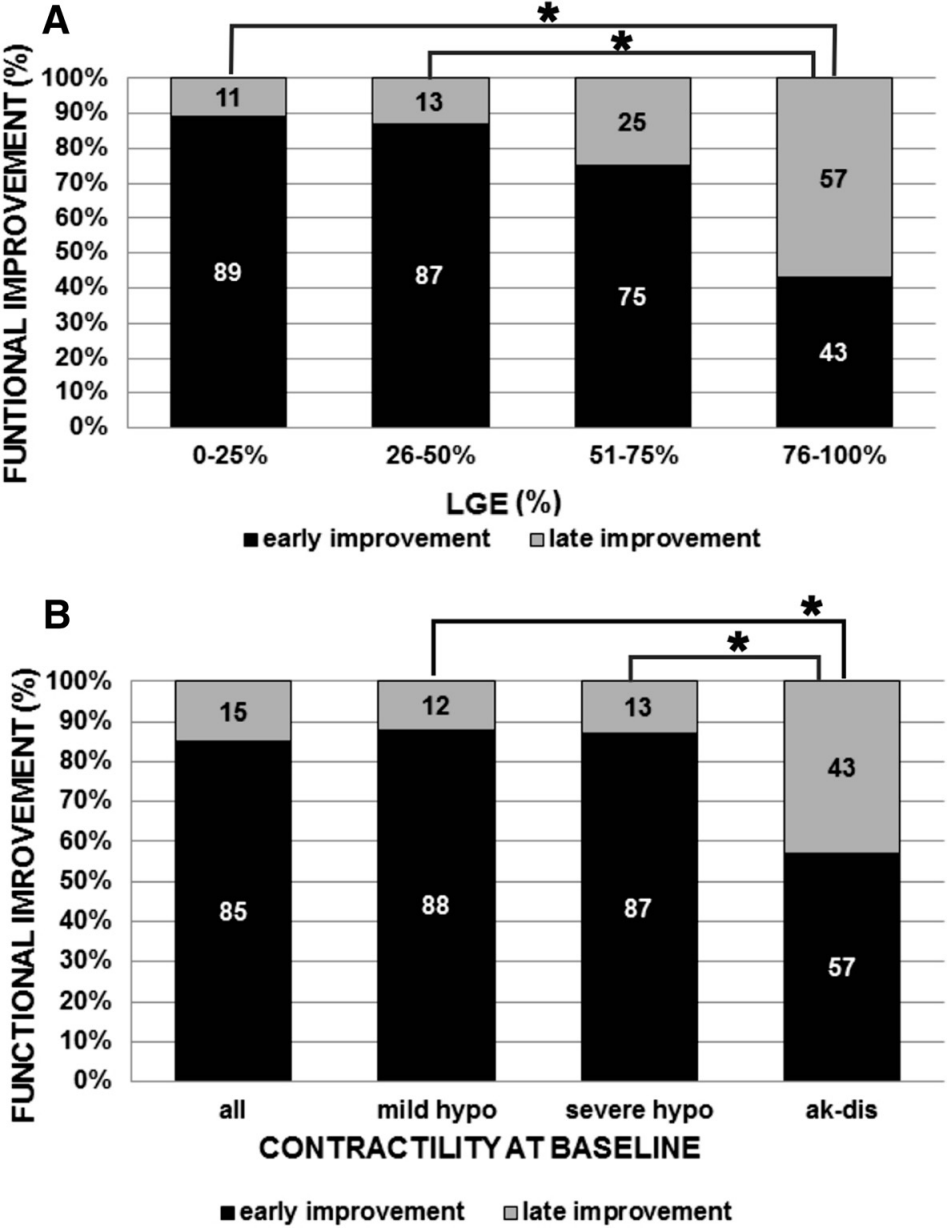
**(A) Time course of segmental functional improvement in relation to LGE at baseline, shown as the relative percentage of improvement at 6 ± 1 months (black bars) and 35 ± 6 months (grey bars) follow-up.** Only segments with functional improvement are included (n = 209); *statistically significant differences between groups. **(B)** Time course of segmental functional improvement in relation to the degree of contractile dysfunction at baseline, shown as the relative percentage of improvement at 6 ± 1 months (black bars) and 35 ± 6 months (grey bars) follow-up. Data are shown for all segments with functional improvement at the end of the study period (first bar, n = 209) and separately for segments with mild hypokinesia (second bar, n = 116), severe hypokinesia (third bar, n = 72) and for segments with akinesia and dyskinesia (fourth bar, n = 21) before revascularisation. *Statistically significant differences between groups.

For the above-mentioned reason we reclassified the segments according to the time of improvement, dividing them into three groups: 1) segments with early (at 6 ± 1 months) improvement, 2) segments with only late (at 35 ± 6 months) improvement, 3) segments without improvement. According to this new classification, the time course of functional improvement was considerably protracted: the majority of the segments (193 of 319, 60.5%) improved in function early, while in a smaller number of segments (32 of 319, 10%) functional improvement was observed only at late follow-up (Figure 2). Although functional improvement continued over the whole study period in all LGE groups, the time course was significantly more delayed in segments with LGE >75% vs. ≤50% LGE at baseline (p = 0.005) (Figure 3A). The majority (96 of 108, 89%) of the improvement in segments with 0–25% LGE was found at 6 ± 1 months, vs. only 43% (3 of 7) with improvement in segments with >75% LGE. Conversely, more than one half (4 of 7, 57%) of the total improvement in segments with >75% LGE occurred between 6 ± 1 months and the final follow-up, vs. only a small fraction (12 of 108, 11%) in segments with 0–25% LGE (Figure 3A).

Although improvement continued over the whole study period in all baseline contractility groups, the time course was slightly more delayed in segments with akinesia and dyskinesia at baseline compared with hypokinesia (p = 0.002) (Figure 3B). The majority (165 of 188, 88%) of the improvement in segments with mild and severe hypokinesia was found early, vs. only 12% (23 of 188) of additional improvement at late follow-up. In contrast, functional improvement in segments with baseline akinesia or dyskinesia was less (12 of 24, 57%) in the first 6 ± 1 months, but at the end of the study period the relative improvement in those segments was greater (9 of 21, 43%) compared with segments with less pronounced dysfunction at baseline (Figure 3B).

The majority (156 of 185, 84%) of segments with CR at baseline improved early, while in a small number of segments (9 of 185, 5%) functional improvement was observed only at late follow-up. Taking into account only the segments with early functional improvement, the majority (156 of 193, 81%) had CR at baseline. In contrast, the majority of those segments (23 of 32, 72%) with only late functional improvement had no CR at baseline. Although improvement continued over the whole study period in both groups (with and without CR), the presence of CR was a predictor of earlier functional improvement (p < 0.0001).

### Global left ventricular systolic function

**Figure 4:**
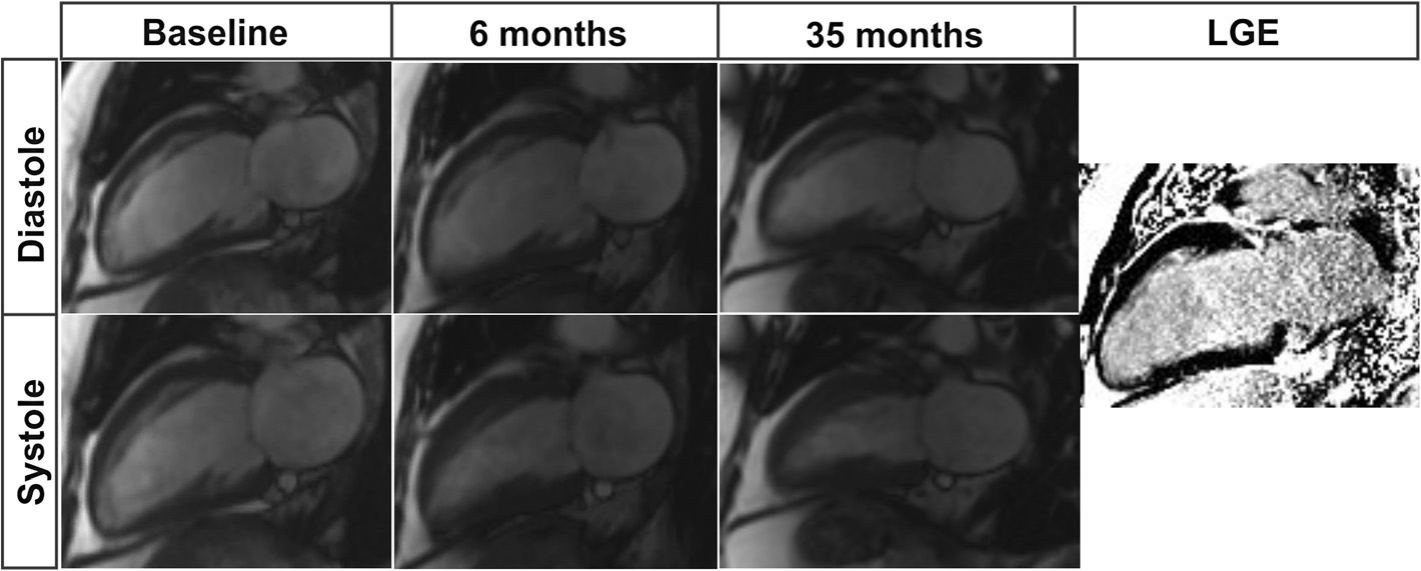
**Example of CMR viability and follow-up studies in a patient with significant improvement in LVEF (52 year old male without previous MI, LVEF at baseline ~24%, and three vessel disease).** There is severe hypokinesia in the anterior and inferior walls at baseline (first column). Six months after revascularisation (complete revascularisation after CABG, 5 distal anastomoses) there is functional recovery in all segments of the anterior and inferior walls (second column), with LVEF ~41%. Thirty-five months after revascularisation the contractile function of the anterior and inferior walls is normal, with LVEF ~48%. There is no LGE in the above-mentioned segments (fourth column).

Overall, the mean improvement in global LV function 35 ± 6 months after revascularisation was 13 ± 10%. Significant LVEF improvement was observed in 32 (76%) patients (an example of a patient with significant improvement in LVEF (responder) is given in Figure 4).

### Changes in LV function

At the end of the study period, significant differences between the responder and nonresponder groups were observed in LVEF (54 ± 11% vs. 35 ± 5%, respectively, p < 0.001), EDV index (EDVI) (85 ± 24 ml/m^2^ vs. 124 ± 31 ml/m^2^, respectively, p = 0.001), ESV index (ESVI) (41 ± 18 ml/m^2^ vs. 82 ± 26 ml/m^2^, respectively, p < 0.001), end-diastolic diameter index (EDDI) (2.6 ± 0.3 cm/m^2^ vs. 3 ± 0.4 cm/m^2^, respectively, p = 0.009), SI 0.55 ± 0.1 vs. 0.63 ± 0.1, respectively, p = 0.016), WMSI (1.4 ± 0.5 vs. 2.1 ± 0.3, respectively, p = 0.001) as well as a significant difference in the number of segments with functional recovery (5.4 ± 3 vs. 2.8 ± 3, respectively, p = 0.009). A significant difference between responder and nonresponder groups was also observed at late follow-up in the mean New York Heart Association (NYHA) functional class (1.3 ± 0.6 vs. 2.2 ± 0.8, respectively, p = 0.002).

Taking into account the changes in LV functional parameters within each group, significant improvements in ESVI, SV, EF, MV RF and WMSI at late follow-up were observed only in the responder group (Table 3). Changes in ESVI, SV, EF and WMSI reached statistical significance in the responder group relatively early (6 months after revascularisation). Meanwhile, more time was needed for a significant decrease in MV RF and additional improvement in LVEF. Changes in SI, EDDI and EDVI didn’t reach statistical significance in either group (Table 3). Taking into account the long-term effect of revascularisation on clinical symptoms in the responder and nonresponder groups, changes in the angina pectoris Canadian Cardiovascular Society (CCS) class (2.9 ± 0.5 to 0.4 ± 0.7, p < 0.0001 vs. 3.0 ± 0 to 1.1 ± 1.2, p = 0.01, respectively) and heart failure NYHA functional class (2.8 ± 0.6 to 1.3 ± 0.6, p < 0.0001 vs. 2.9 ± 0.3 to 2.2 ± 0.8, p = 0.038, respectively) were significant.

**Table 3:** **The dynamic changes in LV function 6 ± 1 and 35 ± 6 months after revascularisation within groups of patients with and without significant improvement in LVEF**

**Responders**	**Baseline**	**6 ± 1 months**	**35 ± 6 months**	Mean diff. ± SD***	***P*** value***
**LV EDVI (ml/m** ^**2**^ **)**	84 ± 34	84 ± 23	85 ± 24	1.6 ± 30	0.94
**LV ESVI (ml/m** ^**2**^ **)**	55 ± 24	43 ± 19*	41 ± 18	14 ± 15	<0.001
**LV SV (ml)**	67 ± 24	83 ± 20*	92 ± 27	24 ± 24	<0.001
**LVEF (%)**	36 ± 8	51 ± 11*	54 ± 11**	17 ± 8	<0.001
**MV RF (%)**	22 ± 11	19 ± 12	16 ± 10	5.0 ± 10	0.021
**WMSI**	1.8 ± 0.4	1.4 ± 0.4*	1.4 ± 0.5	0.4 ± 0.4	<0.001
**Non-responders**					
**LV EDVI (ml/m** ^**2**^ **)**	116 ± 29	109 ± 24	124 ± 31	8.1 ± 28	0.445
**LV ESVI (ml/m** ^**2**^ **)**	77 ± 24	70 ± 19	82 ± 26	4.6 ± 21	0.575
**LV SV (ml)**	77 ± 22	77 ± 18	84 ± 16	7 ± 19	0.359
**LVEF (%)**	33 ± 6	36 ± 8*	35 ± 5	1.3 ± 3.8	0.113
**MV RF (%)**	21 ± 14	19 ± 7	21 ± 13	0.4 ± 18	0.859
**WMSI**	2.1 ± 0.4	1.9 ± 0.3*	2.1 ± 0.3	0.03 ± 0.3	0.779

*LV parameters that differed significantly between the baseline and 6 ± 1 month evaluations; **LV parameters that differed significantly between the 6 ± 1 month and 35 ± 6 month evaluations; ***p values and mean differences ± standard deviation (SD) were calculated for LV parameters assessed at baseline and at the end of the study period. Definitions of the terms and abbreviations are in the text.

### Prediction of significant LVEF improvement

To assess the best CMR-based predictors of significant LVEF improvement, we tested three parameters having significant correlation with changes in LVEF at the end of the study period compared with baseline: the absolute number of viable segments in a patient (r = 0.40, p = 0.008), the percentage of viable segments from all dysfunctional and revascularised segments in a patient (r = 0.56, p < 0.0001) and the number of viable + normal segments in a patient (r = 0.50, p = 0.001). Forward stepwise (Wald) logistic regression analysis was performed to determine the best independent predictor of significant LVEF improvement. This analysis revealed that the percentage of viable segments from all dysfunctional and revascularised segments in a patient was a significant predictor of LVEF improvement ≥5% after revascularisation (p = 0.009). Meanwhile, the absolute number of viable segments in a patient (p = 0.14) and the number of viable + normal segments in a patient (p = 0.37) were not good predictors of significant LVEF improvement. The other LV parameters put into the forward stepwise (Wald) logistic regression analysis, EDVI, ESVI, and WMSI (that differed between the responder and nonresponder groups at baseline) were not good predictors of significant LVEF improvement after revascularisation.

**Figure 5:**
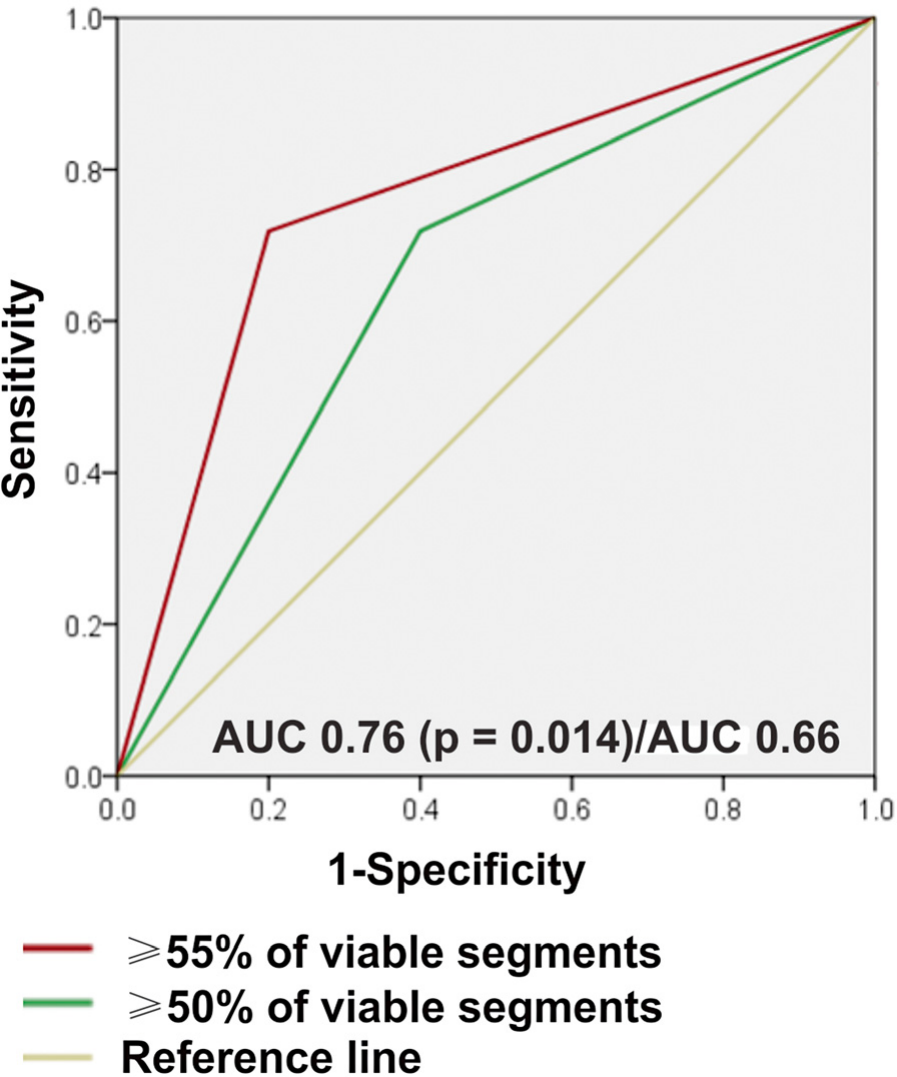
**The areas under the ROC curves for cut-off values of ≥50% (green line) and ≥55% (red line) of viable segments for predicting significant improvement in global LV function after revascularisation.** Definitions of the terms are in the text.

Additionally, using ROC analysis, the AUC for the percentage of viable segments was 0.78 (p = 0.008) compared to AUC 0.63 for the number of viable segments (p = 0.22). An additional ROC analysis was used to define a threshold for the percentage of viable segments in a patient that possessed the optimal sensitivity and specificity for predicting global functional LV recovery. Applying a cut-off of ≥50% viable segments yielded 72% sensitivity and 60% specificity (AUC 0.66, p = 0.132), while a cut-off of ≥55% viable segments yielded 72% sensitivity and 80% specificity (AUC 0.76, p = 0.014) (Figure 5).

## Discussion

This is the first prospective study that used CR and LGE, both assessed by CMR, to predict segmental and global LV functional improvement 35 ± 6 months after revascularisation in patients with chronic ischemic LVD.

### Prediction of segmental functional improvement

The fundamental study by *Kim* et al. [8] was the first to demonstrate a progressive loss of functional recovery with increasing transmural extent of LGE. Furthermore, they demonstrated that although 78% of segments without evidence of scar had improved contractility at 3 months, a significant proportion still did not. Another study by *Bondarenko* et al. [10] found a relatively low improvement rate at 3 months (56% of segments without LGE); however, almost all segments without LGE (93%) showed functional improvement after 24 ± 12 months of follow-up. A study done by *Selvanayagam* et al. [9] reported a high percentage of improved segments (82%) 6 months after surgical revascularisation. Our study showed comparable high percentages of improved segments – 82% of segments without LGE at 6 ± 1 months and 90% of segments without LGE 35 ± 6 months after revascularisation. Performing CMR scans later and excluding patients with procedure-related injury or injury at late follow-up, as we expected, resulted in a high proportion of segments with functional improvement after revascularisation, particularly at the end of the study period. The significant inverse relationship between the likelihood of segmental functional recovery and the LGE or the degree of contractile dysfunction at baseline reported in our study does not contradict the findings of other studies [8-10,13].

We ascertained the predictive accuracy of preprocedural LGE and CR for the recovery of segmental function in a cohort (mean age 65 years with LVEF about 36%, most of the patients with 3 vessel disease and in the NYHA III functional class) typically considered for viability assessment before revascularisation. The definition of a viable segment is based on previous works [11-13] showing that in patients with <75% LGE, the additional assessment of CR by LDD-CMR improves predictive accuracy over LGE imaging alone. For practical purposes we did not complicated this definition by including cut-off values of LGE into the definition of viable segment because, according to our present research, only one segment with LGE >75% (1 of 48, 2%) exhibited CR during LDD-CMR and improved in segmental function at late follow-up. Predicting segmental functional improvement 6 ± 1 months after revascularisation in our cohort and using the above-mentioned definition of viable segment, we obtained sensitivity, specificity, PPV and NPV of 86%, 71%, 82% and 76%, respectively. Predicting segmental functional improvement 35 ± 6 months after revascularisation, we obtained sensitivity, specificity, PPV and NPV of 80%, 70%, 83% and 65%, respectively. Other studies with longer than a 6 month follow-up done by *Bondarenko* at al. [10] (viability criteria was LGE <50%, follow-up 24 ± 12 months) and *Knuesel* et al. [19] (viability criteria was viable rim > 4.5 mm, follow-up 11 ± 2 months) showed slightly lower PPV of 77% and 78% and slightly higher NPV of 73% and 78%, respectively, than our study. Differences in sensitivity, specificity, PPV and NPV obtained using the same viability definition but different follow-up periods in our study is influenced by the prolonged time course of improvement and the 16 segments (5% of all dysfunctional and revascularised segments) that ended up with dysfunction at late follow-up despite improvement 6 months after revascularisation. The addition of LDD-CMR to the LGE-CMR protocol didn’t meaningfully increase the diagnostic accuracy of viability detection compared with other studies that used only LGE-CMR based viability markers [8-11,15,26,27]. The reason for this with other studies with comparable diagnostic accuracy a despite more sophisticated viability assessment protocol and the much longer follow-up time in our study might be the complexity of recovering hibernating myocardium. As we know, the recovery of viable myocardium in the setting of chronic LVD is influenced by a number of additional factors (long-term graft failure or restenosis, duration of hibernation, ongoing LV remodelling, timing of repeated assessment, etc.) and none of the available imaging modalities boast optimal accuracy for predicting regional or global functional improvement [2].

### Time course of functional improvement

The time course of segmental functional improvement in our study was protracted. A comparable percentage of segments continued to show improvement throughout the study period in all LGE groups (Figure 2). However, their relative time courses differed considerably: the time course was significantly more delayed in segments with more extensive LGE (p = 0.005), more severe contractile dysfunction at baseline (p = 0.002) (Figure 3) and the absence of CR at baseline (p < 0.0001). Thus, the presence of CR is a predictor of early functional improvement. The inverse relationship between segmental functional improvement and both baseline LGE and contractile dysfunction could be explained by the fact that more extensive LGE correlates with more severe contractile dysfunction (r = 0.52, p < 0.0001). We found 52% (25 of 48) akinetic or dyskinetic segments in the group with LGE more than 75%; however, only 7% of segments were akinetic or dyskinetic in the group with LGE ≤50%. Similarly, a previous long-term follow-up study done *Bondarenko* at al. [10] suggested that segments with a higher baseline amount of scarring require a longer time to recover. The rationale for this observation is that the degree of degenerative change at the cellular and subcellular levels (e.g., increased extracellular matrix, with replacement of cardiomyocytes by fibrosis) in hibernating myocardium influences the time course of functional improvement [19]. In order for functional improvement to occur it is not the scar *per se* but the unenhanced viable rim that is important – both the thickness and degree of degenerative changes in the viable rim directly influence whether the segment will recover after revascularisation [19,27]. We observed a weak and nonsignificant correlation (r = 0.22, p = 0.128) between the thickness of the unenhanced viable rim and functional improvement in segments with LGE >75%, confirming that not only the thickness but also the degenerative changes in the viable rim are important for functional improvement. As overall improvement in segments with (almost) transmural LGE is low (Figure 3), research studying the subtle morphological changes in the unenhanced viable rim in this LGE group carries more academic than practical value.

### Prediction of global functional improvement

We found significant changes in the mean LVEF after revascularisation, even at late follow-up (13 ± 10%), which suggests that the late improvement in segmental function was sufficient for additional global improvement at the end of the study period despite the 47 segments that ended up with a larger degree of contractile dysfunction compared with baseline. As has been published previously [12], at baseline patients in the nonresponder group (n = 10) had more remodelled left ventricles, lower LVEF, higher LV volume indexes and higher WMSI compared with the responder group (n = 32). At the end of the study period significant difference between the responder and nonresponder groups were observed in LVEF, LV volumes and diameter indexes, SI, WMSI and the number of segments with functional recovery. Thus, patients in the nonresponder group had severely remodelled left ventricles at baseline (ESV 153 ± 46 ml, ESVI 77 ± 24 ml/m^2^) and didn’t show any reverse remodelling at late follow-up (Table 3). Similarly, *Bax* at al. [28] suggested that in patients with substantial viability (≥25% LV), sub-groups with extensive pre-operative remodelling (ESV >140 ml) do not show substantial improvement in LVEF following CABG. Severely dilated LV often do not improve their function after revascularisation, irrespective of the quantity of hibernating myocardium, whilst reverse remodelling following revascularisation is associated with improved prognosis [28-30]. Additionally, subendocardial scarring can prevent systolic thickening at rest, but revascularisation of the mid-myocardial and epicardial layers – which maintains their viability – helps prevent scar expansion [31] and, consequently, even though segmental contractile recovery may not occur, the absence of further cavity dilatation is in fact a benefit of revascularisation [30]. In our study we didn’t find ongoing remodelling in the nonresponder group because changes in LV volumes were insignificant during the entire follow-up. The absence of further LV cavity dilatation together with significant decreases in angina pectoris CCS class and heart failure NYHA functional class may show a benefit of revascularisation in this group. The value of using cut-off values for LV volumes alongside the degree of viability in more remodelled ventricles in order to decide regarding revascularisation should be tested in future studies.

In contrast, patients in the responder group had significant trends of improvement in ESVI, SV, LVEF, and WMSI at the 6 ± 1 month and 35 ± 6 month follow-up. The trend towards a decrease in MV RF was only observed at the end of the study period (p = 0.021). If we define reverse remodelling as ≥15% reduction in the LV end-systolic volume, patients in the responder group experienced reverse remodelling (mean ESV at baseline 112 ± 51 ml vs. 87 ± 40 ml at 6 ± 1 months vs. 83 ± 37 ml at 35 ± 6 months; reduction in mean ESV by ~22% between baseline and 6 ± 1 month follow-up and by ~26% between baseline and 35 ± 6 month follow-up).

Significant improvement in global LV function after revascularisation requires a substantial amount of viable myocardium. Long-term data are scarce, since most CMR-based viability studies have focused on short-term (≤6 months after revascularisation) changes in regional and global function [8,26] or found only small changes in mean global LV function at late follow-up [10]. In our study group, there was a significant positive relation (r = 0.56, p < 0.0001) between the amount of dysfunctional but viable myocardium at baseline, expressed as the percentage of viable segments from all dysfunctional and revascularised segments in a patient, and improvement in the LVEF at late follow-up. As we were basing our experiments on a different study design and relying on our segmental functional recovery prediction results published previously [12], we incorporated LGE-CMR and LDD-CMR data. We analysed different predictors of LVEF improvement ≥5% after revascularisation including the percentage of viable segments [12], the absolute number of viable segments and the number of viable + normal segments in a patient [26]. According to our analysis, the significant independent predictor of LVEF improvement ≥5% after revascularisation at the end of the study was the percentage of viable segments with a cut-off value of ≥55% viable segments, yielding 72% sensitivity and 80% specificity (AUC 0.76, p = 0.014, Figure 5). The predictor of global functional recovery in our study had a lower predictive value than the predictor used in a study conducted by *Pegg* et al. [26]; this could be explained by a difference in the studies’ definitions of significant LVEF improvement (i.e., ≥ 3% change in LVEF [26] versus ≥ 5% change in our cohort) and different follow-up times (i.e., 6 months [26] versus 35 ± 6 months in our study). Interestingly, the percentage of viable segments as a predictor of significant improvement in LVEF reached statistical significance only at the end of the study period, compared with the interim data at 6 months (p = 0.054) [12]. Thus, we think that a cut-off of ≥55% viable segments is a good predictor of global functional improvement, and could be relevant for clinicians making decisions regarding revascularisation in patients with impaired LV function in everyday practice.

### Limitations

The major limitation of the present study is the small sample size. However, this sample size is comparable to previously published studies that used LGE-CMR and LDD-CMR in patients with chronic ischemic LVD undergoing revascularisation [11,13,27]. Additionally, 319 at baseline dysfunctional and successfully revascularised segments were available for statistical analysis, which is more than the required sample size (n = 276) to ensure statistically meaningful predictions of segmental recovery.

In our study, the verification of functional recovery was performed at 6 ± 1 and 35 ± 6 months after revascularisation. The use of two follow-up evaluations for ventricular function with a relatively long period between them may lead to an underestimation of the true rate of functional recovery. The time course of full recovery may be up to 24 ± 12 months [10], and with a longer follow-up period (i.e. 35 months instead of 24 months) some amount of possible recovery could be missed because late graft failure or stent restenosis can negatively affect LV function. Although restenosis/graft occlusion was excluded through invasive procedures at late follow-up in five patients (12%), their non-invasive follow-up revealed that they were free of symptoms or signs indicating recurrent ischemia or major adverse cardiac events. Not one patient from our study group manifested any new LGE zones at late follow-up. The longer follow-up period compared with previous studies represents real clinical practice and gives insights not only into the time course of segmental and global LV functional recovery but also into the long-term sustainability of recovery after revascularisation.

Our study does not take into account that revascularisation does not necessarily result in establishing circulation to all segments, especially when a significantly reduced flow or reduced flow reserve is present in small arterial branches. This is a limitation of our and most other viability studies. By performing myocardial stress perfusion at follow-up is it possible to solve this problem. But then it will be extremely difficult to collect a sufficient sample size and this thoroughly selected cohort won’t represent real clinical practise.

The visual assessment of wall motion is also a limitation of the present study. A quantitative assessment of segmental LV function may be more reliable for assessing subtle changes in contractility, but this approach is rarely used in clinical practice and may not represent the standard viability assessment before revascularisation performed on a daily basis.

## Conclusions

In patients with chronic ischaemic LVD, improvement of dysfunctional but viable myocardium can be considerably delayed. Both the likelihood and the time course of functional improvement are related to the baseline amount of scarring, presence of CR and degree of contractile dysfunction, as visualized by CMR. A cut-off value of ≥55% viable segments from all dysfunctional and revascularised segments in a patient predicts both the long-term significant improvement in LVEF and the reverse LV remodelling. Follow-up CMR scan scheduled 6 months after revascularisation is not long enough to assess the full potential of segmental and global functional recovery. Combination of LDD-CMR and LGE-CMR is a simple and powerful tool for identifying which patients with impaired LV function will benefit from revascularisation.

## Abbreviations

AUC: Area under the curve
CABG: Coronary artery bypass graft surgery
CCS: Canadian Cardiovascular Society
CMR: Cardiovascular magnetic resonance
CR: Contractile reserve
EDDI: End-diastolic diameter index
EDVI: End-diastolic volume index
ECG: Electrocardiogram
ESVI: End-systolic volume index
FOV: Field of view
LDD: Low-dose dobutamine
LGE: Late gadolinium enhancement
LV: Left ventricle
LVEF: Left ventricle ejection fraction
LVD: Left ventricular dysfunction
MI: Myocardial infarction
MV RF: Mitral valve regurgitant fraction
NPV: Negative predictive value
NYHA: New York Heart Association
PCI: Percutaneous coronary intervention
PET: Positron emission tomography
PPV: Positive predictive value
ROC: Receiver operating curve
SI: Sphericity index
SV: Stroke volume
TE: Time of echo
TR: Time of repetition
WMSI: Wall motion score index
